# Label-Free Colorimetric Detection of Influenza Antigen Based on an Antibody-Polydiacetylene Conjugate and Its Coated Polyvinylidene Difluoride Membrane

**DOI:** 10.3390/polym9040127

**Published:** 2017-03-30

**Authors:** Jae-pil Jeong, Eunae Cho, Deokgyu Yun, Taejoon Kim, Im-Soon Lee, Seunho Jung

**Affiliations:** 1 Department of Bioscience and Biotechnology, Microbial Carbohydrate Resource Bank (MCRB), Center for Biotechnology Research in UBITA (CBRU), Konkuk University, 120 Neungdong-ro, Gwangjin-gu, Seoul 05029, Korea; jjp0531@naver.com (J.J.); integrant99@gmail.com (D.Y.); 2 Center for Biotechnology Research in UBITA (CBRU), Institute for Ubiquitous Information Technology and Applications (UBITA), Konkuk University, 120 Neungdong-ro, Gwangjingu, Seoul 05029, Korea; echo@konkuk.ac.kr; 3 Department of Biological Sciences, Konkuk University, 120 Neungdong-ro, Gwangjingu, Seoul 05029, Korea; jusink@konkuk.ac.kr (T.K.); islee@konkuk.ac.kr (I.-S.L.)

**Keywords:** antibody, polydiacetylene, polyvinylidene difluoride, label-free detection, influenza virus

## Abstract

This study presents an antibody-conjugated polydiacetylene (PDA) and its coated polyvinylidene difluoride (PVDF) membrane. The M149 antibody was hybridized to nano-vesicles consisting of pentacosa-10,12-diynoic acid (PCDA) and dimyristoylphosphatidylcholine (DMPC). After photo-polymerization at 254 nm, the effects on the PDA by antigenic injection were investigated with UV-vis spectroscopy, fluorescence spectroscopy, dynamic light scattering and transmission electron microscopy. Because PDA, an alternating ene-yne molecule, induces a blue-to-red color transition and an interesting fluorescent response by the distortion of its backbone, the biomolecular recognition of an antibody–antigen can be converted into an optical and fluorescent signal. Thus, an influenza antigen was successfully detected with the proposed label-free method. Furthermore, the vesicular system was improved by coating it onto a membrane type sensing platform for its stability and portability. The proposed antibody-PDA composite PVDF membrane has potential for rapid, easy and selective visualization of the influenza virus.

## 1. Introduction

Conjugated polymers are composed of alternating single and double bonds, and the overlapping *p*-orbitals give the polymers conductive and emissive properties through the movement of free electrons [1]. Polydiacetylene (PDA), one of the conjugated polymers, is produced by 1,4-addition polymerization of diacetylene monomers using UV irradiation [2]. Under an external stimulation including pH, temperature, and organic solvents, blue PDA undergoes a colorimetric transition to a red color with fluorescence emission [3]. The environmental perturbation induces molecular conformational changes including side chain packing or orientation, and the changed electronic states result in a chromic transition [4]. In addition, compared to small molecule sensors, it has an advantage in amplifying the sensing signal from a molecular recognition. Based on these characteristic properties, its development as unique sensing materials has attracted much attention for environmental and medical applications. For sensor systems, various organic molecules have been functionalized to the PDA domain [5,6,7,8].

Influenza viruses have emerged as a global concern because of their threat to public health and wildlife. In 2009, the influenza A (H1N1) virus spread rapidly all over the world, and the WHO declared a pandemic because of its high infectivity. Recently, South Korea also raised its avian flu alert to maximum as the virus spread. To prevent and control pandemics, the rapid detection of all types of influenza infections is crucial. Thus far, reverse transcription polymerase chain reaction (RT-PCR) and nucleic acid sequence-based amplification (NASBA) have been reported for the diagnosis of influenza [9,10]. Although these methods are highly specific and sensitive, they are time-consuming and expensive. In this respect, other viral detection strategies need to be developed; thus, specific peptide or sialic acid conjugated PDA vesicles have been fabricated based on the recognition of hemagglutinin on the virus surface [11,12].

An antibody (immunoglobulin) is a large Y-shaped protein that specifically recognizes an antigen and is produced by the immune system in response to the presence of an antigen. Conventional immunoassays such as the enzyme-linked immunosorbent assay, fluorescent immunoassay, and radioimmunoassay require labeling strategies with no reduced functionality of the antibody [13]. An antibody modified PDA array can be used as an effective and label-free chromatic immunoassay method. Several antibody-conjugated PDA systems have been used to detect microorganisms [14], secondary antibodies [15], and antigens [16,17].

**Scheme 1:**
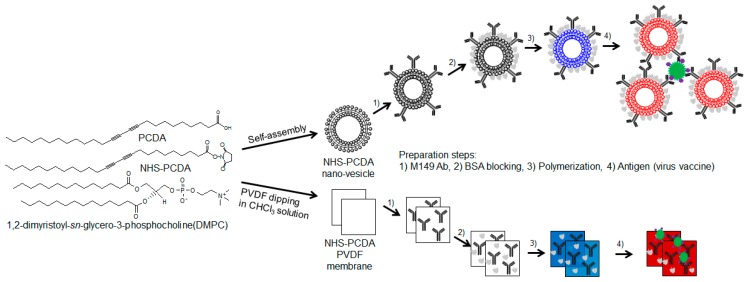
Schematic illustration of the fabrication steps of the M149 antibody-conjugated polydiacetylene (PDA) for influenza A and B virus detection.

Furthermore, the materialization of PDA-based sensors opens the possibility of the development of commercially available detection kits. In particular, paper-based sensors have advantages because they are flexible, disposable, inexpensive, and portable [18]. Additionally, the oxidative damage and aging shown in vesicle types can be overcome [19,20]. This study for the first time presents M149 antibody functionalized PDA platforms in the form of nano-vesicles and membrane strips to detect virus antigens (Scheme 1). The PDA nano-vesicles before and after adding the virus antigens were examined by UV-vis spectroscopy, fluorescence spectroscopy, dynamic light scattering and transmission electron microscopy. With a polyvinylidene difluoride (PVDF) membrane, sample handling and cost efficiency can be improved.

## 2. Materials and Methods

### 2.1. Materials

The 10,12-Pentacosadiynoic acid (PCDA) was purchased from Sigma Aldrich (St. Louis, MO, USA). The 1,2-Dimyristoyl-*sn*-glycero-3-phosphocholine (DMPC) was purchased from Avanti Polar lipids (Alabaster, AL, USA). *N*-hydroxysuccinimide (NHS) was purchased from Fluka (Geneva, Switzerland). The 1-(3-Dimethylaminopropyl)-3-ethyl carbodiimide hydrochloride (EDC) was purchased from Acros organics (Morris Plains, NJ, USA). Magnesium sulfate anhydrous was purchased from Shinyo pure chemicals (Osaka, Japan). Bovine Serum Albumin (BSA) was purchased from Merck (Novagen, Germany). Rabbit anti-human influenza A and B virus polyclonal antibody (M149) was purchased from Takara (Tokyo, Japan). SKY Cell Flu Quadrivalent (SK chemical, Andong, Korea) consists of four influenza strains [A/Christchurch/16/2010, NIB-74xp(H1N1); A/HongKong/4801/2014, NYMC,X-263(H3N2); B/Brisbane/60/2008, NYMC,BX-35; B/Phuket/3073/2013]. The vaccine suspension has 15 μg/mL of purified inactivated split virus particles from each of the four virus strains. PVDF transfer membrane (0.45 µm) was purchased from EMD Millipore (Billerica, MA, USA).

### 2.2. Synthesis of the N-Hydroxysuccinimide Ester of 10,12-Pentacosadiynoic (NHS-PCDA)

First, 1 g of PCDA (2.7 mmoL) was dissolved in 10 ml of dichloromethane. To the solution, NHS (337.5 mg, 2.9 mmol) and EDC (615 mg, 3.2 mmoL) were added [21]. The resulting organic solution was stirred for 4 h at room temperature. After removing the solvent in vacuo, the product was extracted using ethyl acetate and water. Then, magnesium sulfate anhydrate was added to the organic layer. After the organic solvent part was filtered, the solvent was removed in vacuo. A white colored product was obtained at 710 mg. The product was confirmed with thin-layer chromatography (TLC, hexane/ethyl acetate 3:1) and ^1^H NMR spectroscopy (see the Supplementary data, Figure S1).

### 2.3. Preparation of NHS-PCDA Nano-Vesicles

The three lipid molecules were dissolved in chloroform at the desired molar ratios (NHS-1: NHS-PCDA 10%, PCDA 50%, DMPC 40%, NHS-2: NHS-PCDA 20%, PCDA 40%, DMPC 40%, NHS-3: NHS-PCDA 30%, PCDA 30%, DMPC 40%) for a total of 1 mM of lipid. Chloroform was removed by flowing N_2_ gas, and a thin lipid film was obtained on the glass surface. HEPES buffer solution (pH 8, 5 mM) was added to give a total lipid concentration of 1 mM. The samples were heated at 80 °C for 15 min. and sonicated for 12.5 min. using a probe sonicator (Sonics VC-505, Newtown, PA, USA) at 40% power. The warm solution was filtered through a 0.8 μm cellulose acetate filter (Advantec, Tokyo, Japan) to remove any undispersed lipid, and the resulting milky solution was cooled to 4 °C overnight [22].

### 2.4. Conjugation of the M149 Antibody to the NHS-PCDA Nano-Vesicles

A mixture of 1 mM NHS-PCDA vesicle solution (1 mL) and M149 antibody (1 μg/mL) was incubated at 4 °C overnight. Any remaining NHS groups were blocked by reacting with the amine groups of BSA (150 μg/mL) in HEPES buffer for 1 h at 4 °C. The antibody modified vesicle solution was polymerized by UV irradiation for 5 min. [23].

### 2.5. Colorimetric Response (CR%) Measurement of NHS-1, NHS-2 and NHS-3 PCDA Nano-Vesicles

The degree of color change for the PDA vesicles was characterized by the CR%, which was calculated with the following equation:

$$\mathrm{CR}\%=\frac{PB_{b}-PB_{a}}{PB_{b}}\times 100\%$$

where $PB=A_{\mathrm{blue}}/\left(A_{\mathrm{blue}}+A_{\mathrm{red}}\right)$, $A_{\mathrm{blue}}$ is the absorbance at 650 nm for the blue phase, and $A_{\mathrm{red}}$ is the absorbance at 550 nm for the red phase. $PB_{a}$ and $PB_{b}$ are the PB values after and before the addition of the virus vaccine.

### 2.6. Fluorescence Spectroscopy

Because the blue-to-red color change of the PDAs occurs along with the generation of fluorescence, the fluorescence intensity was observed by a fluorescence microplate reader (Molecular Devices, Sunnyvale, CA, USA) at room temperature. The fluorescence spectra were measured after the addition of the virus vaccine for 10 min. The excitation wavelength was 485 nm.

### 2.7. Transmission Electron Microscopy (TEM)

The vesicles were loaded onto a Formvar-coated copper grid (200 mesh) and dried. A 2% uranyl acetate solution was added to the sample. After washing and drying, the nano-vesicles were then examined with energy-filtering TEM (LIBRA 120, Carl Zeiss, Germany).

### 2.8. Dynamic Light Scattering (DLS)

DLS measurements were done with a Dynamic Plate Reader (Wyatt Technology, Santa Barbara, CA, USA) to assess the size distribution of the vesicles.

### 2.9. Performance of the Immunoreactions for Vaccine Detection

The purified influenza vaccines were diluted to a final concentration of 3.3, 6.6, 16.5 and 33 μg/mL in HEPES buffer (TCI CHemical, Tokyo, Japan), respectively. The vaccines were added to the polymerized antibody-conjugated PDA vesicles and incubated at room temperature for 10 min. HEPES buffer was added to the antibody-conjugated vesicles as a negative control. The color of the vesicle solution was observed with the naked eye, and the absorption spectrogram of the solution was recorded for a wavelength from 450 to 750 nm with a 1 mm optical path length on a UV-2450 UV-vis spectrophotometer (Shimadzu, Kyoto, Japan).

### 2.10. Preparation of the Sensor Membranes for Virus Antigen Detection

A solution of PCDA–NHS (0.3 mmoL), PCDA (1.5 mmoL), and DMPC (1.2 mmoL) was prepared in 15 mL chloroform. The PVDF membrane was cut into strips using scissors. The membranes were dipped into the diacetylene solution and then dried in a fume hood. The membranes were incubated in a solution containing 1 μg/mL of polyclonal M149 antibody overnight at 4 °C. Next, the membranes were incubated in 150 μg/mL of BSA for 1 h to inactivate any remaining NHS sites, rinsed with deionized water, and allowed to dry in the dark. Photoirradiation of the membranes was done with a LF-206.LS UV hand lamp (UVItec, Cambridge, UK) for 5 min generating a blue color. After adding the antigen, fluorescence microscopic images were obtained by an Olympus BX61-32FDIC upright fluorescence microscope (Olympus, Tokyo, Japan).

### 2.11. Scanning Electron Microscopy (SEM)

The original and modified PVDF membranes were observed with SEM (Jeol, JSM 6380, Tokyo, Japan) after being sprayed with gold.

## 3. Results

### 3.1. Composition of the Antibody-PDA Conjugate

**Figure 1:**
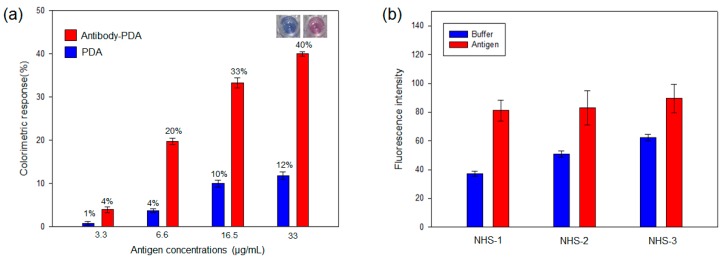
(**a**) Colorimetric response (CR%) of the antibody-PDA conjugates and unfunctionalized PDA against antigen concentrations. Inset shows the noticeable color change of antibody-PDA conjugate after the addition of virus antigen (33 µg/mL); (**b**) Fluorescence intensity of the antibody-PDA conjugates in the absence and presence of virus antigen. (NHS-1 = 10% NHS-PCDA, NHS-2 = 20% NHS-PCDA and NHS-3 = 30% NHS-PCDA) The virus antigen was added to the PDA system and incubated at 37 °C for 10 min.

The NHS ester of PCDA is made to create reactive acylating agents, and the reaction with the amine groups of antibodies produces the conjugate of the antibody-PDA. In the present work, the M149 antibody was coupled with PCDA and DMPC mixed vesicles using NHS ester-mediated acylation. For enhanced fluidity and sensitivity without interrupting the chromatic properties of the PDA, 40% DMPC was distributed within the conjugated PDA [16]. To optimize the composition, the antibody-PDA conjugates were fabricated with three different NHS-PCDA ratios (10%, 20% and 30%). In the antibody-PDA conjugates, the blue-to-red color transition was clearly observed after adding the virus antigen; however, the unmodified PDA vesicles showed low and saturated CR% values against antigen concentrations (Figure 1a and Figure S1). This result indicates that the molecular recognition of the antibody–antigen was successfully expressed as a color shift, whereas, nonspecific electrostatic interactions might induce the small increase in CR% [24] In succession, the fluorescence intensities were measured for the three modified PDA systems, and the 10% NHS-PCDA showed the largest fluorescent enhancement (Figure 1b). The fluorescence in the absence of the antigen was due to the incubation at 37 °C and the bulkiness of the modified group. For PDA-based sensing platforms, 10% modified PCDA has also been used in other reports [25,26]. Taken together, the NHS-1 composition was selected to detect the viral antigens.

### 3.2. Visible Spectroscopic Monitoring of the Color Transition against Antigen Concentrations

**Figure 2:**
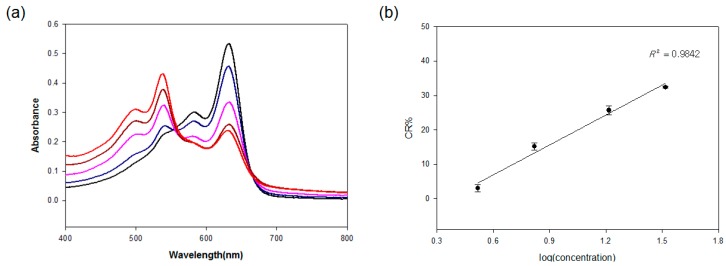
(**a**) Visible spectroscopy for different antigen concentrations (0 (**black**), 3.3 (**blue**), 6.6 (**pink**), 16.5 (**brown**), 33 µg/mL (**red**)); (**b**) Correlation curve between the CR% and log (concentration of the virus antigen). All experiments were performed in triplicate.

To confirm the red-phase transition of the antibody-conjugated PDA, the visible spectroscopic behavior was further investigated (Figure 2a). The PDA showed an absorption maximum of 650 nm and a weaker absorption at 550 nm, rendering it a blue color that is detectable by the naked eye. Upon addition of the antigen to the antibody-conjugated PDA vesicles, the absorbance at 650 nm decreased with a concurrent increase of the absorbance appearing at 550 nm, and a color change was observed from blue to red. For the quantitative results of the color change, the respective CR% was calculated and plotted against the antigen concentrations (Figure 2b). The present antibody-PDA conjugate showed a good linear relationship for the increasing concentrations of the antigen in the range of 3.3 to 33 µg/mL.

### 3.3. Immunological Detection of the Antibody-PDA Conjugate Nano-Vesicles

**Figure 3:**
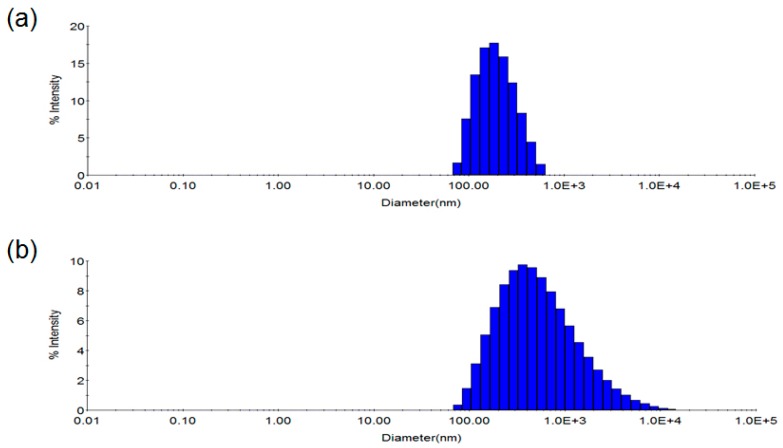
Dynamic light scattering (DLS) profile of the antibody-conjugated PDA nano-vesicles. (**a**) Without virus antigen; (**b**) With virus antigen.

To gain insight into the mechanism for this chromatic transition, the size distribution of the antibody-conjugated PDA vesicles before and after antigen binding was compared with DLS (Figure 3). The average diameter of the original antibody-PDA conjugate was measured as 215 nm. Upon the addition of the viral antigens, the DLS profile of the red shifted vesicles was broadened, and the average size could not be detected. This result represents a disruption, fusion or aggregation of the antibody-PDA vesicles in the presence of the influenza vaccine.

**Figure 4:**
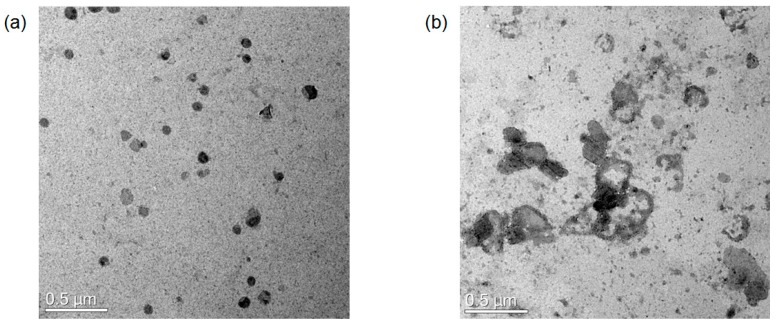
TEM images of the antibody-conjugated PDA nano-vesicles. (**a**) Without virus antigen; (**b**) With virus antigen.

To confirm our assumption, TEM image analyses were performed. Figure 4 shows the morphological change of the antibody-PDA conjugates before and after the addition of the virus antigen. Antibody conjugated PDA vesicles have relatively round shapes with around 100 nm in general, whereas the shapes of the antigen added sample exist in an aggregated and fused form. Some vesicles also showed a burst morphology with an irregular shell. The discrepancy with the DLS size can be attributed to the solid and swollen vesicles in the TEM and DLS, respectively [27]. These results show the invisible physical shifts beyond the optical changes after adding the antigen to the antibody-conjugated PDA vesicles. Therefore, it is suggested that the binding events on the surface of the PDA array lead to vesicle disruption and reduced conjugation, causing the absorption of the shorter wavelength; thus, red light can be shown.

### 3.4. PVDF Membranes Coated with the Antibody-PDA Conjugates

**Figure 5:**
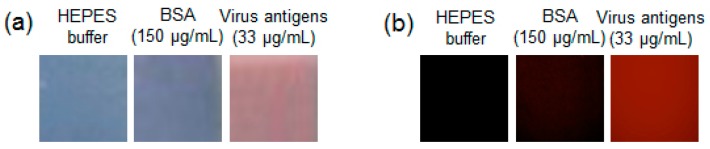
(**a**) Optical and (**b**) fluorescent microscopic images of the antibody-PDA functionalized polyvinylidene difluoride (PVDF) membranes.

**Figure 6:**
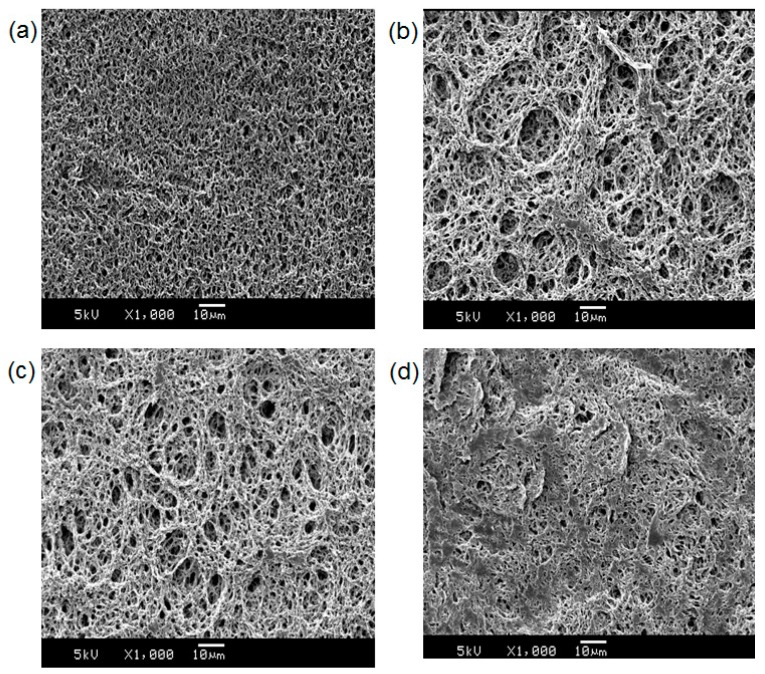
SEM images of the (**a**) original PVDF; (**b**) buffer-treated antibody-PDA coated PVDF; (**c**) BSA-treated antibody-PDA coated PVDF; and (**d**) virus antigen-treated antibody-PDA coated PVDF.

For materialization, PDA based sensing membranes were fabricated as described in the materials and methods. The antibody-PDA coated PVDA membranes were dipped into a solution of HEPES buffer, BSA, and virus antigens. After incubation at 37 °C for 30 min., the virus antigens produced not only a blue-to-red color change but also red fluorescence (Figure 5). As a negative control, a five-fold concentration of BSA was used; however, no significant change was observed. The chemical composition of the membranes was investigated using FT-IR analysis. The FT-IR spectra are shown in Figure S3 and the peaks are assigned in Table S1. Compared to the original PVDF membrane, some new peaks were observed in antibody-PDA coated PVDF membranes because of protein components. Furthermore, the morphological changes of the membrane surfaces were observed by SEM (Figure 6). After antibody-PDA coating, the membrane fibers grew thicker, the network structure was more complex, and the pore size was varied. It is attributed to the coverage of the antibody-PDA layer [28]. The addition of virus antigen further changes the coated PVDF membrane, and the flattened surface is seen with decreased pore size and number. This result can be explained for the antibody–antigen interaction, considering that the porous adsorbent surface is filled with adsorption materials via non-covalent interaction [29].

## 4. Conclusions

In summary, we report here an antibody-PDA conjugate and its PVDF membrane for influenza virus detection. The M149 antibody was conjugated to PCDA/DMPC vesicles by NHS ester-mediated acylation. After photopolymerization, the developed conjugated polymers were examined for colorimetric and fluorescent detection of the virus vaccine. To further improve the stability and portability, its coated PVDF membrane system was fabricated and well applied. It is supposed that the molecular recognition between the hemagglutinin on the virus surface and the antibody-PDA conjugates contributes to the present chromatic immunoassay. This study provides a strategy for the development of chemosensing materials based on biopolymer-conjugated polymers.

## Supplementary Materials

The following are available online at www.mdpi.com/2073-4360/9/4/127/s1, Figure S1: Colorimetric responses (%) for various antigen concentrations in the antibody-PDA conjugates and unfunctionalized PDA, Figure S2: DLS profile of non-modified PDA nano-vesicles (average diameter = 175 nm), Figure S3: FT-IR spectra of the original PVDF (blue line), buffer-treated antibody-PDA coated PVDF (purple line), BSA-treated antibody-PDA coated PVDF (green line), and virus antigen-treated antibody-PDA coated PVDF (red line), Table S1: FT-IR absorption bands of N-benzyltriazole derivatized dextran before and after adsorption.

## References

[1] McQuade D.T., Pullen A.E., Swager T.M. (2000). Conjugated polymer-based chemical sensors. Chem. Rev..

[2] Wegner G. (1969). Topochemische reaktionen von monomeren mit konjugierten dreifachbindungen/tochemical reactions of monomers with conjugated triple bonds. Zeitschrift für Naturforschung B.

[3] Charych D.H., Nagy J.O., Spevak W., Bednarski M.D. (1993). Direct colorimetric detection of a receptor-ligand interaction by a polymerized bilayer assembly. Science.

[4] Potisatityuenyong A., Rojanathanes R., Tumcharern G., Sukwattanasinitt M. (2008). Electronic absorption spectroscopy probed side-chain movement in chromic transitions of polydiacetylene vesicles. Langmuir.

[5] Lee K.M., Chen X., Fang W., Kim J.M., Yoon J. (2011). A dual colorimetric and fluorometric sensor for lead ion based on conjugated polydiacetylenes. Macromol. Rapid Commun..

[6] Yun D., Cho E., Dindulkar S.D., Jung S. (2016). Succinoglycan octasaccharide conjugated polydiacetylene-doped alginate beads for barium (II) detection. Macromol. Mater. Eng..

[7] Ma G., Cheng Q. (2005). Vesicular polydiacetylene sensor for colorimetric signaling of bacterial pore-forming toxin. Langmuir.

[8] Rangin M., Basu A. (2004). Lipopolysaccharide identification with functionalized polydiacetylene liposome sensors. J. Am. Chem. Soc..

[9] Suarez D.L., Das A., Ellis E. (2007). Review of rapid molecular diagnostic tools for avian influenza virus. Avian Dis..

[10] Jernigan D., Lindstrom S., Johnson J., Miller J., Hoelscher M., Humes R., Shively R., Brammer L., Burke S., Villanueva J. (2011). Detecting 2009 pandemic influenza a (H1N1) virus infection: Availability of diagnostic testing led to rapid pandemic response. Clin. Infect. Dis..

[11] Lim E.-K., Guk K., Kim H., Chung B.-H., Jung J. (2015). Simple, rapid detection of influenza a (H1N1) viruses using a highly sensitive peptide-based molecular beacon. Chem. Commun..

[12] Reichert A., Nagy J.O., Spevak W., Charych D. (1995). Polydiacetylene liposomes functionalized with sialic acid bind and colorimetrically detect influenza virus. J. Am. Chem. Soc..

[13] Ishikawa E., Imagawa M., Hashida S., Yoshitake S., Hamaguchi Y., Ueno T. (1983). Enzyme-labeling of antibodies and their fragments for enzyme immunoassay and immunohistochemical staining. J. Immunoass. Immunochem..

[14] De Oliveira T.V., de FF Soares N., Coimbra J.S.D.R., de Andrade N.J., Moura L.G., Medeiros E.A., de Medeiros H.S. (2015). Stability and sensitivity of polydiacetylene vesicles to detect salmonella. Sens. Actuators B Chem..

[15] Kim K.-W., Choi H., Lee G.S., Ahn D.J., Oh M.-K., Kim J.-M. (2006). Micro-patterned polydiacetylene vesicle chips for detecting protein-protein interactions. Macromol. Res..

[16] Su Y.-L., Li J.-R., Jiang L. (2004). Chromatic immunoassay based on polydiacetylene vesicles. Colloids Surf. B Biointerfaces.

[17] Jiang L., Luo J., Dong W., Wang C., Jin W., Xia Y., Wang H., Ding H., Jiang L., He H. (2015). Development and evaluation of a polydiacetylene based biosensor for the detection of H5 influenza virus. J. Virol. Methods.

[18] Liana D.D., Raguse B., Gooding J.J., Chow E. (2012). Recent advances in paper-based sensors. Sensors.

[19] Du Plessis J., Ramachandran C., Weiner N., Müller D. (1996). The influence of lipid composition and lamellarity of liposomes on the physical stability of liposomes upon storage. Int. J. Pharm..

[20] Hernández-Caselles T., Villalain J., Gómez-Fernández J. (1990). Stability of liposomes on long term storage. J. Pharm. Pharmacol..

[21] Cho E., Kim H., Yang J.E., Jun B.-H., Paik S.R., Jung S. (2014). Supramolecular self-assembled aggregates formed by pentacosa-10,12-diynyl amidomethyl-β-cyclodextrin. Carbohydr. Res..

[22] Yun D., Jeong D., Cho E., Jung S. (2015). Colorimetric detection of some highly hydrophobic flavonoids using polydiacetylene liposomes containing pentacosa-10,12-diynoyl succinoglycan monomers. PLoS ONE.

[23] Kang D.H., Jung H.-S., Lee J., Seo S., Kim J., Kim K., Suh K.-Y. (2012). Design of polydiacetylene-phospholipid supramolecules for enhanced stability and sensitivity. Langmuir.

[24] Kolusheva S., Boyer L., Jelinek R. (2000). A colorimetric assay for rapid screening of antimicrobial peptides. Nat. Biotechnol..

[25] Wang D.-E., Wang Y., Tian C., Zhang L., Han X., Tu Q., Yuan M., Chen S., Wang J. (2015). Polydiacetylene liposome-encapsulated alginate hydrogel beads for Pb 2^+^ detection with enhanced sensitivity. J. Mater. Chem. A.

[26] Okada S., Peng S., Spevak W., Charych D. (1998). Color and chromism of polydiacetylene vesicles. Acc. Chem. Res..

[27] Sun T., Zhang H., Kong L., Qiao H., Li Y., Xin F., Hao A. (2011). Controlled transformation from nanorods to vesicles induced by cyclomaltoheptaoses (β-cyclodextrins). Carbohydr. Res..

[28] Zhu L.-P., Yu J.-Z., Xu Y.-Y., Xi Z.-Y., Zhu B.-K. (2009). Surface modification of PVDF porous membranes via poly (DOPA) coating and heparin immobilization. Colloids Surf. B Biointerfaces.

[29] Cho E., Tahir M.N., Kim H., Yu J.-H., Jung S. (2015). Removal of methyl violet dye by adsorption onto *n*-benzyltriazole derivatized dextran. RSC Adv..

