# Supplementary material for: Label-Free Colorimetric Detection of Influenza Antigen Based on an Antibody-Polydiacetylene Conjugate and Its Coated Polyvinylidene Difluoride Membrane
Source: Polymers (Basel). 2017 Mar 30;9(4):127. doi: 10.3390/polym9040127 (PMC6432067; doi:10.3390/polym9040127)
Supplement: Supplementary file 1 [file polymers-09-00127-s001.pdf]

Supplementary Materials: Label-Free Colorimetric Detection of Influenza Antigen Based on an Antibody-Polydiacetylene Conjugate and Its Coated Polyvinylidene Difluoride Membrane

Jae-pil Jeong, Eunae Cho, Taejoon Kim, Im-Soon Lee and Seunho Jung

1. NMR Spectroscopy

For the NMR spectroscopic analysis, a Bruker Avance 500 spectrometer (Bruker, Billerica, MA, USA) was used to record the ^{1}H NMR spectrum.

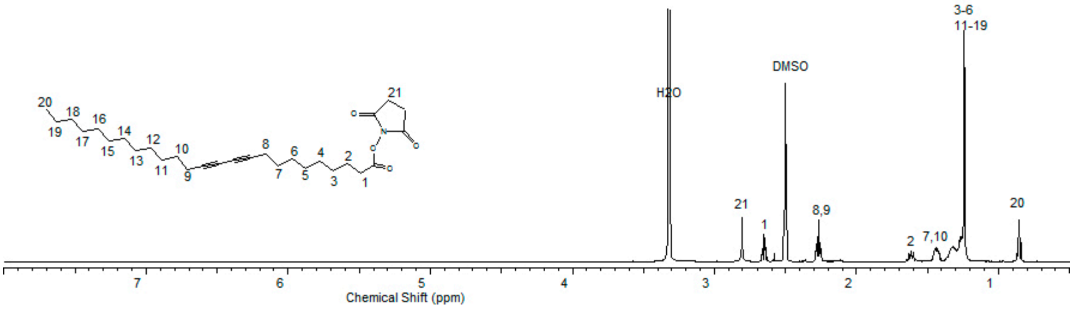

Figure S1. ^{1}H-NMR spectrum (in DMSO-d_6) of NHS-PCDA. NHS-PCDA. ^{1}H NMR (500 MHz, DMSO-d_6): δ 2.81(m, 4H, 21), 2.65 (t, 2H, 1), 2.27 (t, 4H, 8,9), 1.61 (t, 2H, 2), 1.48 (br, 4H, 7,10), 1.40–1.22 (br, 26H, 3–6,11–19), 0.85 (t, 3H, 20).

Preparation of Unfunctionalized PDA Vesicles

The two lipid molecules were dissolved in chloroform at the desired molar ratios (PCDA 60%, DMPC 40%) for a total of 1 mM of lipid. Chloroform was removed by flowing N_2 gas, and a thin lipid film was obtained on the glass surface. HEPES buffer solution (pH 8, 5 mM) was added to give a total lipid concentration of 1 mM. The samples were heated at 80 °C for 15 min. and sonicated for 12.5 min. using a probe sonicator (Sonics VC-505, Newtown, PA, USA) at 40% power. The warm solution was filtered through a 0.8 μm cellulose acetate filter (Advantec, Tokyo, Japan) to remove any undispersed lipid, and the resulting milky solution was cooled to 4 °C overnight. After photopolymerization, the resulting PDA vesicles were used to compare with antibody conjugated PDA vesicles.

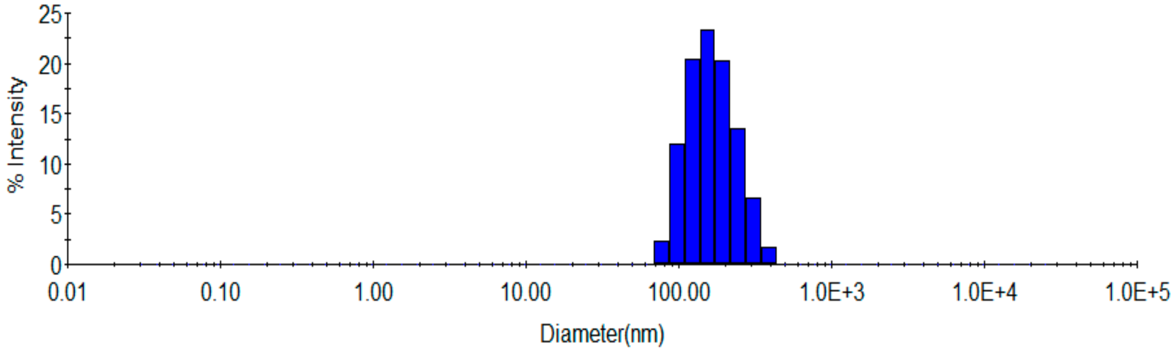

Figure S2. DLS profile of non-modified PDA nano-vesicles (average diameter = 175 nm).

2. Fourier-Transform Infrared (FT-IR) Spectroscopy

FT-IR spectra were obtained in ATR mode using a Nicolet iS50 spectrometer (Thermo Nicolet Instrument Corporation, Madison, WI, USA).

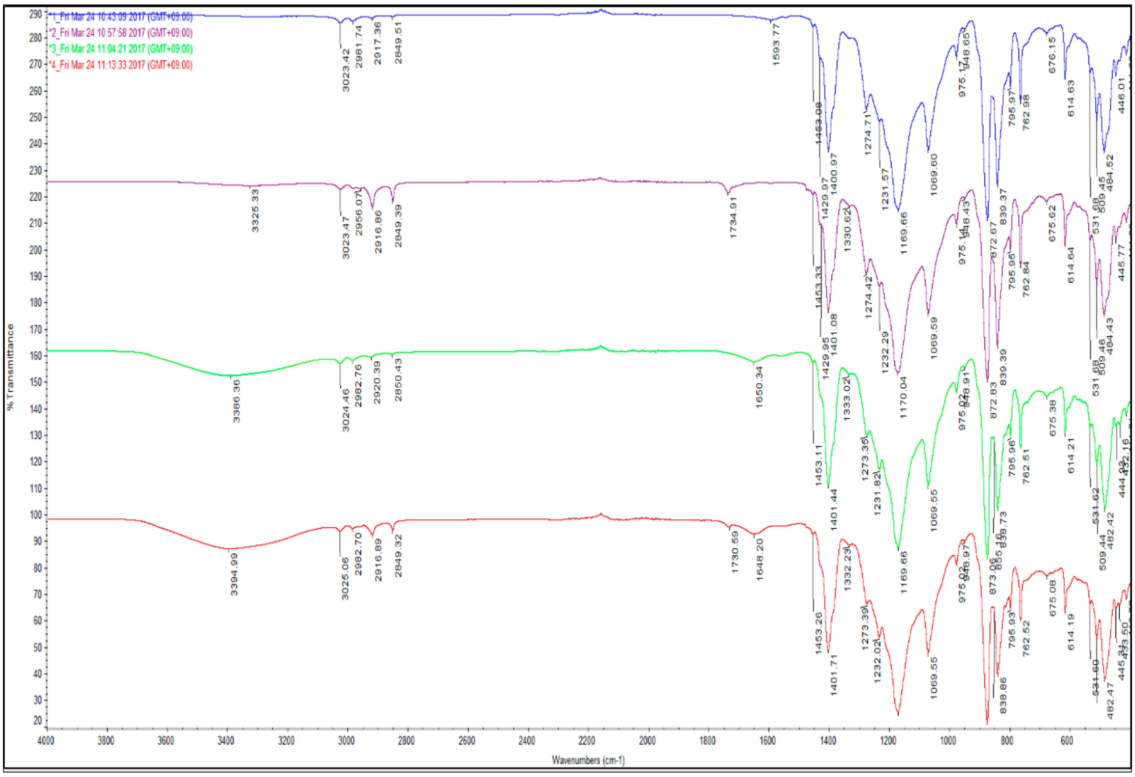

Figure S3. FT-IR spectra of the original PVDF (**blue line**), buffer-treated antibody-PDA coated PVDF (**purple line**), BSA-treated antibody-PDA coated PVDF (**green line**), and virus antigen-treated antibody-PDA coated PVDF (**red line**).

Table S1. FT-IR absorption bands of *N*-benzyltriazole derivatized dextran before and after adsorption.

Wavenumber (cm^{-1})				Assignment
**Original PVDF**	**Buffer-treated antibody-PDA coated PVDF**	**BSA-treated antibody-PDA coated PVDF**	**Virus antigen-treated antibody-PDA coated PVDF**	
	3325	3386	3395	O–H stretch
3023	3023	3024	3025	Asymmetric CH_2 stretch
2982	2956	2983	2983	Symmetric CH_2 stretch
	1735		1731	Carboxylic acid C=O stretch
		1690	1648	Amide C=O stretch
1401	1402	1401	1402	CH_2 wagging, C–C–C asymmetric stretch
1232	1232	1232	1232	C–F asymmetric stretch
1170	1170	1170	1170	C–C, CH_2 stretch
873	873	873	873	C–C–C asymmetric stretch, CF_2 asymmetric stretch
839	839	839	839	CH_2 rocking, CF_2 asymmetric stretch
